# Clinical features of *Escherichia coli* positive calcium oxalate stones and effects of outer membrane vesicles on tubular epithelial injury and crystal adhesion

**DOI:** 10.3389/fcimb.2026.1859506

**Published:** 2026-08-20

**Authors:** Xijie Ding, Qingchen Du, Xinyang Niu, Endi Zhang, Jianxing Li, Guojun Chen, Chaoyue Ji, Weiguo Hu

**Affiliations:** 1 Department of Urology, Qinghai University Affiliated Hospital, School of Clinical Medicine, Qinghai University, Xining, China; 2 Department of Urology, Beijing Tsinghua Changgung Hospital, School of Clinical Medicine, Tsinghua Medicine, Tsinghua University, Beijing, China

**Keywords:** calcium oxalate stone, crystal adhesion, *Escherichia coli*, outer membrane vesicles, oxidative stress

## Abstract

**Background:**

Although calcium oxalate (CaOx) stones are classically regarded as a metabolic stone type, bacterial factors may also contribute to clinical heterogeneity. However, the biological relevance of bacteriuria in CaOx stone disease remains unclear. We investigated the clinical association between bacteriuria and CaOx stone phenotype and examined whether Escherichia coli-derived outer membrane vesicles (OMVs) could induce epithelial changes relevant to crystal retention.

**Methods:**

We retrospectively analyzed 1,976 patients with urinary stones treated at a single center between 2016 and 2020, including 1,277 with CaOx stones, 465 with infection stones, and 234 with uric acid stones. Patients with CaOx stones were further stratified by urine culture status and bacterial species. In parallel, OMVs were isolated from a uropathogenic E. coli reference strain and characterized by transmission electron microscopy and nanoparticle tracking analysis. Their effects on HK-2 cells were assessed using confocal internalization assays, CCK-8, reactive oxygen species detection, oxidative stress-related biochemical assays, and calcium oxalate monohydrate (COM) crystal adhesion assays.

**Results:**

CaOx, infection, and uric acid stones exhibited distinct clinical characteristics and different measured urinary biochemical parameters. Within the CaOx cohort, urine culture-positive patients had larger stones and a higher proportion of renal stones than culture-negative patients despite lower 24-hour urinary excretion of several ions. Among patients with culture positive CaOx stones, those positive for *E. coli* were characterized by older age, female predominance, renal stone predominance, larger maximum stone diameter, and lower urinary excretion of several ions measured over 24 hours, whereas the pooled other bacteria group showed a less uniform pattern. *E. coli*-derived OMVs were internalized by HK-2 cells in a time-dependent manner, reduced cell viability, increased reactive oxygen species and malondialdehyde, decreased superoxide dismutase and catalase activity, and enhanced COM crystal adhesion.

**Conclusion:**

Clinical and experimental findings suggest that *E. coli*-associated bacterial signals may be relevant to a subset of CaOx stone disease and provide biological plausibility for *E. coli*-derived OMVs as cell-free bacterial effectors that may link bacteriuria to tubular epithelial injury and crystal retention.

## 1 Introduction

Kidney stone disease imposes a substantial health burden, with an estimated prevalence of 13% in men and 7% in women in the United States (Malieckal et al., 2023), and recurrence reported in up to 67% of first-time stone formers within 5 years (D'Costa et al., 2019). Calcium oxalate (CaOx) stones account for approximately 80% of all kidney stones and represent the predominant metabolic subtype of nephrolithiasis (Wang et al., 2024). Their formation is classically linked to lithogenic alterations in urinary chemistry, particularly hypercalciuria, hyperoxaluria, and hypocitraturia, which promote urinary supersaturation and intraluminal crystal formation (Khan et al., 2021). Together, these observations indicate that CaOx nephrolithiasis is fundamentally a metabolically driven stone disease. However, growing evidence suggests that metabolic abnormalities alone may not fully explain the heterogeneity of CaOx stone formation, and that additional non-metabolic factors may also contribute to its pathogenesis.

Among the potential non-metabolic factors implicated in CaOx stone disease, bacteria have attracted increasing attention. Culture-based evidence suggests that bacterial involvement is not limited to classical struvite calculi. In a surgical cohort of 153 patients, da Cruz Machado et al (da Cruz MaChado et al., 2024). reported that, among culture-positive non-struvite stones, 45.5% were composed of calcium oxalate monohydrate, and 86.4% of these culture-positive non-struvite stones yielded non-urease-producing bacteria. Consistently, Zhong et al (Zhong et al., 2021). investigated Escherichia coli isolated from urine and stone cultures in 66 patients with CaOx stones and collected 83 isolates, including 33 from urine and 50 from stones, showing that E. coli predominated in both urine and stone cultures and that paired isolates from the same patient were highly similar in antimicrobial susceptibility, phylogenetic grouping, virulence genes, and genomic relatedness. Experimental studies further support a biologically relevant role of bacteria in CaOx stone formation. Chutipongtanate et al (Chutipongtanate et al., 2013). showed that viable bacteria, including *E. coli*, could promote CaOx crystal growth and aggregation *in vitro*, and Kanlaya et al (Kanlaya et al., 2019). further demonstrated that *E. coli* flagellum enhanced CaOx crystallization, crystal growth, aggregation, and crystal-cell adhesion. Together, these findings support a biologically relevant association between E. coli and CaOx stone disease; however, most available mechanistic studies have focused on intact bacteria or bacterial surface-associated structures.

A key mechanistic question therefore remains unresolved: whether cell free bacterial effectors may also contribute to E. coli associated CaOx stone disease. Outer membrane vesicles (OMVs), nanoscale vesicles released by Gram-negative bacteria, represent a biologically plausible candidate because they can be internalized by host cells and deliver lipopolysaccharides, proteins, and other bioactive cargo independently of direct bacterial contact (Dean et al., 2020). We therefore hypothesized that E. coli-derived OMVs may induce renal tubular epithelial injury and crystal adhesion, two epithelial phenotypes relevant to CaOx stone formation. To test this hypothesis, we designed the clinical analysis as a stepwise stratification rather than as a single broad comparison. First, we compared CaOx, infection, and uric acid stones to define the clinical features and measured urinary biochemical parameters of CaOx stones relative to two major non-CaOx phenotypes. Second, within the CaOx cohort, we examined whether urine culture positivity was associated with stone location, maximum stone diameter, and measured urinary biochemical parameters, which were selected as clinically relevant indicators of the anatomical setting of crystal retention and selected aspects of the urinary lithogenic environment. Third, because Escherichia coli was the most frequent organism among culture-positive CaOx cases and has been linked to CaOx stones in previous studies, we further explored whether E. coli-positive patients showed a recognizable clinical profile compared with culture-negative patients and patients with other bacterial isolates. We then used E. coli-derived OMVs as a representative cell-free bacterial effector to investigate whether they could be internalized by renal tubular epithelial cells and induce stone-relevant epithelial phenotypes *in vitro*.

## 2 Materials and methods

### 2.1 Patients and study design

A retrospective analysis was conducted on consecutive patients with urinary stones who underwent percutaneous nephrolithotomy or flexible ureteroscopy at our institution between January 2016 and December 2020. Intraoperative stone specimens were collected and analyzed for stone composition by Fourier transform infrared spectroscopy. Calcium oxalate (CaOx) stones were defined as stones containing ≥50% calcium oxalate monohydrate and/or calcium oxalate dihydrate. Infection stones were defined as stones containing ≥50% magnesium ammonium phosphate hexahydrate and/or carbonate apatite. Uric acid (UA) stones were defined as stones containing >50% uric acid (Skolarikos et al., 2024).

Urine culture results obtained at admission were collected for all patients. Positive urine cultures were defined as bacterial growth >10^5^ CFU/mL in women and >10^4^ CFU/mL in men (Chen et al., 2025). A total of 2,144 patients were initially identified. After excluding patients with incomplete stone composition data or incomplete clinical records, 1,976 patients were included in the final analysis. Patients were first categorized according to stone composition into the CaOx, infection stone, and UA stone groups. Patients with CaOx stones were further stratified according to urine culture status as urine culture-positive and urine culture-negative groups. For additional subgroup analyses, patients with CaOx stones were further classified into Escherichia coli, other bacterial isolates, and culture-negative groups according to admission urine culture results. Clinical data collected included age, sex, body mass index (BMI), comorbidities, stone characteristics, urinalysis, urine culture results at admission, stone composition, and 24-hour urinary biochemical parameters, as presented in the tables of this study. Because 24-hour urinary oxalate and citrate were not routinely measured at our institution during the study period, these parameters were not available in this retrospective dataset.

### 2.2 Bacterial culture

To improve experimental consistency and reproducibility while retaining relevance to urinary infection, we used E. coli CFT073/WAM2267 (ATCC 700928), a genome sequenced uropathogenic E. coli (UPEC) reference strain reported as serotype O6:K2:H1. CFT073 was originally isolated from blood and urine from a woman with acute pyelonephritis. Its complete genome is available in public databases, including GenBank accession AE014075.1, RefSeq accession NC_004431.1, and assembly accession GCA_000007445.1/GCF_000007445.1 (Welch et al., 2002). E. coli ATCC 700928 was purchased from Ningbo Mingzhou Biotechnology Co., Ltd. (Ningbo, China) and grown in Luria–Bertani (LB) broth (Solarbio, Beijing, China) at 37 °C with shaking. Bacterial growth was monitored by measuring optical density at 600 nm, and cultures were harvested at the mid-logarithmic phase (OD600 = 0.6) for outer membrane vesicle (OMV) isolation (Wang et al., 2025).

### 2.3 Isolation and purification of *Escherichia coli* outer membrane vesicles

OMVs were isolated from *E. coli* culture supernatants using sequential centrifugation, membrane filtration, and OptiPrep density-gradient ultracentrifugation (Hong et al., 2019; Zhang et al., 2024). After removal of residual bacteria and debris by centrifugation and filtration through 0.45- and 0.22-μm membranes (Merck Millipore, Darmstadt, Germany), crude vesicles were pelleted by ultracentrifugation at 100,000 × g for 2 h at 4 °C in polycarbonate bottles (Beckman Coulter, Brea, CA, USA). To minimize contamination by bacterial debris, membrane fragments, and other non-vesicular components, the pellets were further purified on a discontinuous 10%–40% OptiPrep gradient prepared with iodixanol solution (Serumwerk Bernburg AG, Bernburg, Germany) at 100,000 × g for 4 h at 4 °C. OMV-enriched fractions (15%–25% OptiPrep) were collected (Hong et al., 2019), washed by ultracentrifugation, resuspended in sterile PBS, stored at −80 °C, and quantified using a micro BCA protein assay kit (Regen Biotechnology, Beijing, China).

### 2.4 Characterization of OMVs by TEM and NTA

To confirm vesicle morphology and size distribution, purified OMVs were analyzed by TEM and NTA. For TEM, OMVs were deposited onto copper grids, negatively stained with 2% uranyl acetate, and observed with a Hitachi H-7650 transmission electron microscope (Hitachi, Japan) at 80 kV. For NTA, samples were diluted in PBS to 1 × 10^7–1 × 10^9 particles/mL and measured using a ZetaView PMX 110 analyzer (Particle Metrix, Germany) with a 405-nm laser.

### 2.5 Cell culture and OMV treatment

HK-2 cells were purchased from Procell (Wuhan, China) and cultured in RPMI-1640 medium containing 10% fetal bovine serum (Gibco, USA) and 1% penicillin/streptomycin (Procell, Wuhan, China) at 37 °C with 5% CO_2_. Unless otherwise indicated, HK-2 cells grown to 60%–70% confluence were treated with *Escherichia coli*-derived OMVs at the specified concentrations and durations (Hu et al., 2020).

### 2.6 Cell counting kit-8 assay

To evaluate the effect of OMVs on cell viability, HK-2 cells were seeded in 96-well plates at 5 × 10^3^ cells per well and treated with *Escherichia coli*-derived OMVs (0, 50, 75, or 100 μg/mL) for 3, 6, 12, or 24 h. Cell viability was measured using an Enhanced Cell Counting Kit-8 (Bioss) following the manufacturer’s protocol. Absorbance was recorded with a SUNRISE microplate reader (Tecan, Austria), and results were normalized to the untreated control group. The concentration and duration used for subsequent functional assays were selected based on this CCK-8 concentration- and time-response experiment. Because urinary OMV concentrations in patients with CaOx stones have not been established, these *in vitro* doses were intended to provide a reproducible epithelial injury model rather than to reproduce an exact urinary exposure level.

### 2.7 OMV labeling and internalization assay

To determine whether OMVs could be internalized by HK-2 cells, purified *Escherichia coli*-derived OMVs were labeled with PKH67 green fluorescent dye (Sigma, MINI67) with minor modifications of established protocols (Jiang et al., 2024; Zhang et al., 2024). After incubation of 10 μg OMVs with PKH67 for 30 min at room temperature in the dark, the labeling reaction was terminated with 1% BSA in PBS. To exclude nonspecific fluorescence caused by free PKH67 dye or dye aggregates, a dye-only control was prepared using the same labeling, quenching, and washing procedures in the absence of OMVs. HK-2 cells seeded in glass-bottom confocal dishes (1 × 10^5^ cells/dish) were exposed to PKH67-labeled OMVs or dye-only control for 3, 6, or 12 h, followed by fixation with 4% paraformaldehyde and counterstaining with DAPI (Beyotime, China) and phalloidin (Beyotime, China). Fluorescence images were acquired using a Nikon A1R laser scanning confocal microscope (Nikon, Japan). PKH67-positive area was quantified using ImageJ from randomly selected fields, and data were obtained from three independent experiments.

### 2.8 Measurement of intracellular reactive oxygen species

Intracellular ROS generation was assessed using a DCFH-DA Reactive Oxygen Species Assay Kit (Beyotime Biotechnology, Shanghai, China) according to the manufacturer’s instructions. HK-2 cells were treated with *Escherichia coli*-derived OMVs (100 μg/mL) for 12 h, followed by incubation with 10 μM DCFH-DA at 37 °C for 20 min in the dark. Fluorescence images were acquired using an EVOS M7000 imaging system (Thermo Fisher Scientific, USA), and fluorescence intensity was quantified with ImageJ.

### 2.9 Measurement of MDA, SOD, and CAT

To evaluate oxidative stress, malondialdehyde (MDA) content and the activities of superoxide dismutase (SOD) and catalase (CAT) were measured in HK-2 cells using commercial assay kits according to the manufacturers’ instructions. After treatment with *Escherichia coli*-derived OMVs (100 μg/mL) for 12 h, cell lysates were collected for biochemical analysis. SOD activity, MDA content, and CAT activity were determined using WST-8–based, lipid peroxidation, and catalase assay kits, respectively (all from Beyotime Biotechnology, Shanghai, China). Absorbance was measured using a SUNRISE microplate reader (Tecan, Austria), and results were normalized to total protein concentration determined by BCA assay.

### 2.10 Calcium oxalate monohydrate crystal adhesion assay

To assess crystal adhesion, HK-2 cells were grown in 6-well plates to 60%–70% confluence and pretreated with *Escherichia coli*-derived OMVs (100 μg/mL) for 12 h. The medium was then replaced, and calcium oxalate monohydrate (COM) crystals (3A, Anhui Sunrise Technology Co., Ltd., Anhui, China; 98% purity) were added at a final concentration of 100 μg/mL for 6 h. After gentle washing with PBS to remove non-adherent crystals, cells were examined under an inverted light microscope (Leica DMi1, Leica Microsystems, Germany). Crystal adhesion was quantified from randomly selected fields using ImageJ and expressed as the percentage of crystal area relative to cell area (Xu et al., 2025).

### 2.11 Statistical analysis

Statistical analyses were performed separately for the clinical and *in vitro* experimental data. For the clinical analyses, continuous variables were first tested for normality using the Kolmogorov–Smirnov test and for homogeneity of variance using Levene’s test. Normally distributed data are presented as mean ± standard deviation (SD), whereas non-normally distributed data are presented as median (interquartile range, IQR). Comparisons among the calcium oxalate (CaOx), infection stone, and uric acid (UA) groups, as well as among the *Escherichia coli*, other bacterial isolates, and culture-negative subgroups within CaOx stones, were performed using one-way analysis of variance (ANOVA) followed by Bonferroni-adjusted *post hoc* comparisons when variances were equal, or Welch’s ANOVA when variances were unequal. Non-normally distributed variables were compared using the Kruskal–Wallis test followed by Dunn’s *post hoc* test where appropriate. For comparisons between urine culture-positive and urine culture-negative patients with CaOx stones, continuous variables were analyzed using the independent-samples Student’s *t* test or Mann–Whitney *U* test, as appropriate. Categorical variables are presented as frequencies and percentages and were compared using the chi-square test or Fisher’s exact test, followed by Bonferroni-corrected pairwise comparisons when required. For pairwise comparisons among three groups, a Bonferroni-corrected *P* value < 0.0167 was considered statistically significant. To further evaluate whether urine culture status was associated with major host and stone factors within the CaOx cohort, multivariable logistic regression analyses were performed. In the first model, urine culture positivity was used as the dependent variable, with urine culture negative patients as the reference group. In the additional models, E. coli positive urine culture and other bacteria positive urine culture were separately compared with negative urine culture. Age, sex, BMI, hypertension, diabetes, stone laterality, stone location, and maximum stone diameter were included as covariates. Age and maximum stone diameter were modeled per 10 year and per 10 mm increase, respectively. Results were reported as adjusted odds ratios (aORs) with 95% confidence intervals (CIs). These models were intended to evaluate adjusted associations rather than to establish causal direction.

For the *in vitro* experiments, data are presented as mean ± SD from at least three independent experiments unless otherwise indicated. Image-based quantification was performed using ImageJ. Comparisons between two groups were analyzed using the unpaired two-tailed Student’s *t* test. For experiments involving more than two groups, one-way ANOVA followed by Tukey’s or Bonferroni multiple-comparison test was used. For CCK-8 experiments involving both OMV concentration and treatment time, two-way ANOVA was used when appropriate. Unless otherwise specified, a two-sided *P* value < 0.05 was considered statistically significant. In figures, statistical significance was indicated as follows: P < 0.05 (*), P < 0.01 (**), and P < 0.001 (***). Clinical data were analyzed using SPSS version 26.0 (IBM Corp., Armonk, NY, USA), and experimental data were analyzed using GraphPad Prism 9.0 (GraphPad Software, San Diego, CA, USA).

## 3 Results

### 3.1 Clinical characteristics differed among patients with CaOx, infection, and UA stones

We first compared CaOx, infection, and uric acid stones to determine whether CaOx stones in this cohort showed distinct clinical features and measured urinary biochemical parameters compared with classical infection-associated stones and uric acid stones. This comparison was intended to provide the phenotypic context for subsequent analyses focused specifically on CaOx stone disease. A total of 1,976 patients were included in the clinical analysis, including 1,277 with calcium oxalate (CaOx) stones, 465 with infection stones, and 234 with uric acid (UA) stones. Significant differences were observed among the three groups in demographic features, comorbidities, renal biochemical indices, stone characteristics, urine culture status, and 24-hour urinary parameters (**Table 1**).

**Table 1:** Comparison of clinical characteristics among patients with calcium oxalate, infection, and uric acid stones.

Characteristic	CaOx stones	Infection stones	UA stones	P-value
Patients	1277	465	234	
Age	50.5 ± 13.7 ^a, b^	48.2 ± 13.9 ^c^	54.0 ± 12.9	<0.001
Sex				<0.001
Male(n)	889 (69.6%) ^a^	180 (38.7%) ^c^	174 (74.4%)	
Female(n)	388 (30.4%)	285 (61.3%)	60 (25.6%)	
Body Mass Index (kg/m²)	25.27 ± 4.19 ^a, b^	24.19 ± 4.51 ^c^	26.51 ± 3.88	<0.001
Underlying diseases				
Hypertensive	386 (30.2%) ^a, b^	84 (18.1%) ^c^	115 (49.1%)	<0.001
Diabetes	195 (15.3%) ^b^	57 (12.3%) ^c^	54 (23.1%)	<0.001
Biochemical testing				
Creatinine (μmol/L)	89.47 ± 70.67 ^a, b^	101.60 ± 80.48 ^c^	126.52 ± 91.10	<0.001
Uric Acid (μmol/L)	367.43 ± 96.62 ^a, b^	353.36 ± 106.70 ^c^	432.57 ± 115.56	<0.001
eGFR (mL/min/1.73 m²)	90.32 ± 37.58 ^a, b^	83.84 ± 36.14 ^c^	70.83 ± 32.75	<0.001
Parathyroid Hormone (pg/mL)	58.80 ± 70.20	60.38 ± 46.23	64.30 ± 53.54	0.519
Serum Calcium (mmol/L)	2.26 ± 0.15 ^a^	2.23 ± 0.14 ^c^	2.25 ± 0.12	<0.001
Stone character				
Stone laterality				<0.001
Unilateral	971 (76.0%) ^a, b^	309 (66.5%) ^c^	67 (28.6%)	
Bilateral	306 (24.0%)	156 (33.5%)	167 (71.4%)	
Location of stones				<0.001
Renal	882 (69.1%) ^a^	402 (86.5%) ^c^	176 (75.2%)	
Ureteral	232 (18.2%)	20 (4.3%)	25 (10.7%)	
Renal and Ureteral	163 (12.8%)	43 (9.2%)	33 (14.1%)	
Maximum stone diameter (mm)	25.53 ± 19.53 ^a, b^	48.29 ± 33.57 ^c^	30.89 ± 20.28	<0.001
Urine culture				<0.001
Positive	487 (38.1%) ^a^	308 (66.2%) ^c^	98 (41.9%)	
Negative	790 (61.9%)	157 (33.8%)	136 (58.1%)	
24-hour urinalysis				
Urine pH	6.25 ± 0.55 ^a, b^	6.68 ± 0.62 ^c^	5.67 ± 0.65	<0.001
Volume (L/D)	2.04 ± 0.77 ^a^	2.26 ± 0.90 ^c^	1.96 ± 0.79	<0.001
Chlorine (mmol/D)	68.61 ± 31.99 ^a^	55.85 ± 25.84 ^c^	73.10 ± 35.00	<0.001
Sodium (mmol/D)	84.85 ± 37.46 ^a^	69.15 ± 31.04 ^c^	89.60 ± 38.87	<0.001
Potassium (mmol/D)	17.16 ± 8.09 ^a^	14.18 ± 6.60 ^c^	18.70 ± 10.51	<0.001
Calcium (mmol/D)	2.17 ± 1.24 ^a, b^	1.53 ± 1.11	1.74 ± 1.43	<0.001
Phosphorus (mmol/D)	9.12 ± 5.54 ^a^	6.42 ± 4.04 ^c^	9.81 ± 5.23	<0.001
Uric acid (μmol/D)	1570.93 ± 764.16 ^a^	1272.89 ± 667.50 ^c^	1689.09 ± 839.56	<0.001

Superscript letters indicate significant pairwise differences after Bonferroni correction: a, CaOx stones vs infection stones; b, CaOx stones vs UA stones; c, infection stones vs UA stones (all *P < 0.0167*).

Compared with the other groups, patients with infection stones were younger and more frequently female, whereas patients with UA stones were older and predominantly male. Body mass index was highest in the UA stone group and lowest in the infection stone group. The prevalence of hypertension and diabetes also differed significantly across groups. In biochemical testing, the UA stone group showed the highest serum creatinine and serum uric acid levels and the lowest eGFR, whereas the CaOx group had relatively more preserved renal function. Parathyroid hormone levels did not differ significantly among groups.

Stone characteristics also varied markedly among groups. Infection stones were associated with the largest maximum stone diameter, with a mean maximum diameter of 48.29 ± 33.57 mm, compared with 25.53 ± 19.53 mm in the CaOx group and 30.89 ± 20.28 mm in the UA group. Bilateral stones were most frequent in the UA stone group, whereas renal stones predominated in the infection stone group. Urine culture positivity was highest in the infection stone group (66.2%), compared with 38.1% in the CaOx group and 41.9% in the UA group. In addition, the measured 24-hour urinary parameters differed significantly among groups, including urine pH, urine volume, chloride, sodium, potassium, calcium, phosphorus, and uric acid excretion. Overall, these results indicate that CaOx, infection, and UA stones represent clinically distinct phenotypes with different profiles of measured urinary biochemical parameters.

### 3.2 Microbial distributions varied across urine culture-positive stone subtypes

**Figure 1:**
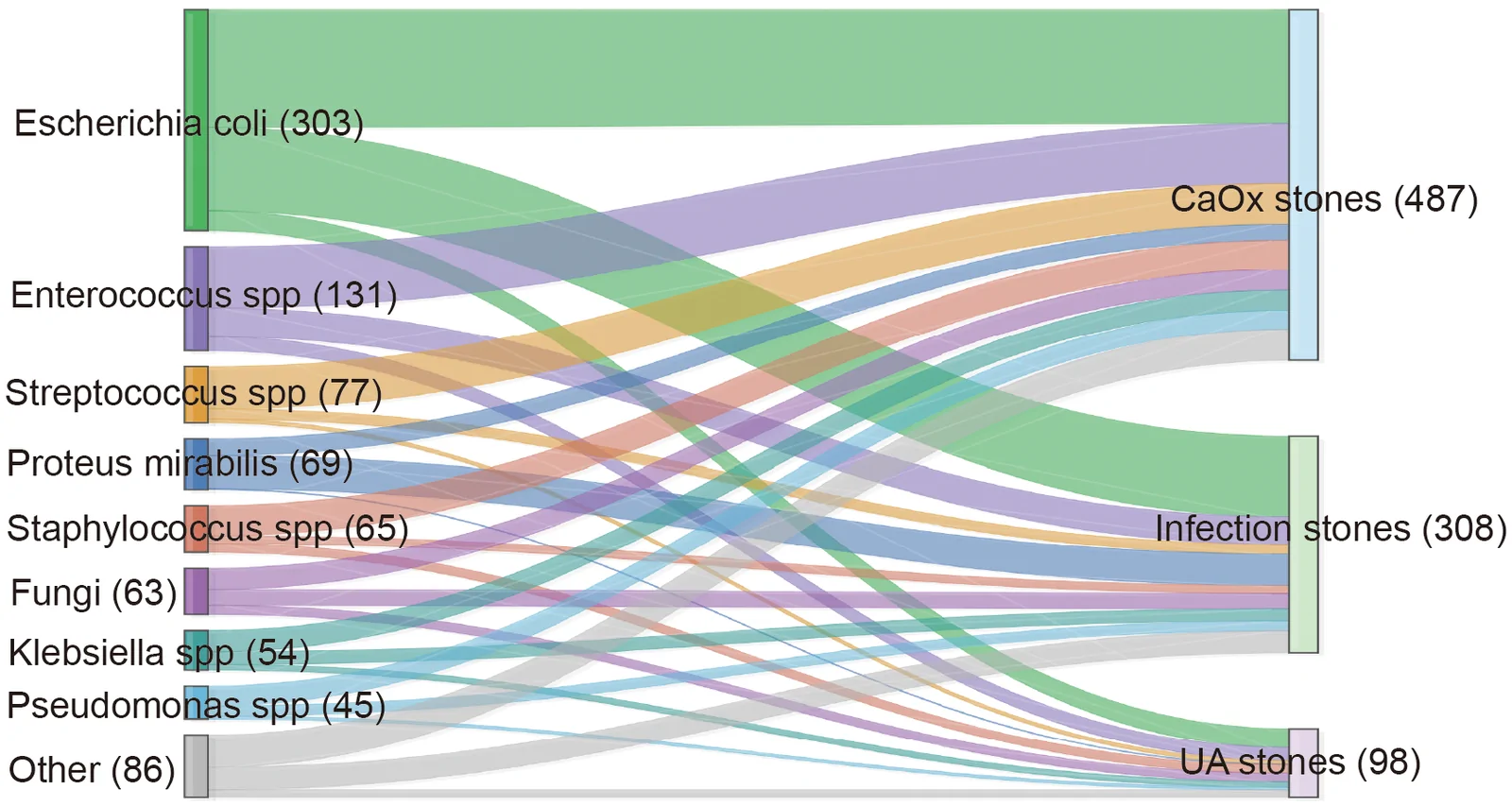
Sankey diagram showing the distribution of microorganisms identified in urine culture-positive patients with calcium oxalate (CaOx) stones, infection stones, and uric acid (UA) stones.

To further visualize bacteriological differences among stone types, a Sankey diagram was constructed for urine culture-positive patients with CaOx, infection, and UA stones (**Figure 1**). The diagram showed that the spectrum of microorganisms differed among stone categories, supporting heterogeneity in bacteriological composition across different stone types.

### 3.3 Urine culture positivity was associated with distinct stone characteristics and measured urinary biochemical parameters in CaOx stones

After establishing the overall clinical context of CaOx stones, we next focused on the CaOx cohort to examine whether urine culture positivity was associated with stone location, maximum stone diameter, and measured urinary biochemical parameters in this conventionally metabolic stone type. The primary features of interest were stone location, maximum stone diameter, and 24-hour urinary biochemical parameters, because these variables may reflect the anatomical setting of crystal retention and selected aspects of the urinary lithogenic environment. Among the 1,277 patients with CaOx stones, 487 were urine culture-positive and 790 were urine culture-negative. Compared with culture-negative patients, culture-positive patients were older (52.1 ± 14.4 vs 49.5 ± 13.1 years, *P* = 0.01) and more frequently female. They also showed a different stone distribution, with a higher proportion of renal stones and a larger maximum stone diameter (29.20 ± 22.84 vs 23.36 ± 16.91 mm, *P* < 0.001) (**Table 2**). In serum biochemical analyses, culture-positive CaOx patients had lower serum uric acid levels than culture-negative patients, while serum creatinine, eGFR, parathyroid hormone, and the prevalence of hypertension or diabetes did not differ significantly. In 24-hour urine analyses, culture-positive patients had a slightly higher urine pH and lower sodium, calcium, phosphorus, and uric acid excretion. Chloride and potassium excretion were also reduced in the culture-positive group. These findings suggest that, within CaOx stone disease, the presence of urinary bacteria is associated with distinct clinical features and differences in measured urinary biochemical parameters.

**Table 2:** Comparison of clinical characteristics between urine culture-positive and urine culture-negative patients with calcium oxalate stones.

Characteristic	Urine culture-positive CaOx stones	Urine culture-negative CaOx stones	P-value
Patients	487	790	
Age	52.1 ± 14.4	49.5 ± 13.1	0.01
Sex			<0.001
Male	256 (52.6%)	633 (80.1%)	
Female	231 (47.4%)	157 (19.9%)	
Body Mass Index (kg/m²)	25.36 ± 4.79	25.21 ± 3.77	0.536
Underlying Diseases			
Hypertensive	159 (32.6%)	227 (28.7%)	0.139
Diabetes	81 (16.6%)	114 (14.4%)	0.288
Biochemical testing			
Creatinine (μmol/L)	90.63 ± 71.22	88.75 ± 70.36	0.644
Uric Acid (μmol/L)	352.30 ± 94.42	376.96 ± 96.84	<0.001
eGFR (mL/min/1.73 m²)	88.72 ± 52.44	91.32 ± 24.13	0.230
Parathyroid Hormone (pg/mL)	61.98 ± 86.15	56.87 ± 58.42	0.260
Serum Calcium (mmol/L)	2.25 ± 0.16	2.27 ± 0.14	0.019
Stone character			
Stone laterality			
Unilateral	359 (73.7%)	612 (77.5%)	0.127
Bilateral	128 (26.3%)	178 (22.5%)	
Location of stones			<0.001
Renal	369 (75.8%)	513 (64.9%)	
Ureteral	55 (11.3%)	177 (22.4%)	
Renal and Ureteral	63 (12.9%)	100 (12.7%)	
Maximum stone diameter (mm)	29.20 ± 22.84	23.36 ± 16.91	<0.001
24-hour urinalysis			
Urine pH	6.34 ± 0.49	6.20 ± 0.58	0.041
Volume (L/D)	2.10 ± 0.83	1.99 ± 0.73	0.051
Chlorine (mmol/D)	65.69 ± 30.77	70.69 ± 32.70	0.025
Sodium (mmol/D)	81.09 ± 35.68	87.49 ± 38.47	0.015
Potassium (mmol/D)	16.47 ± 7.90	17.64 ± 8.20	0.037
Calcium (mmol/D)	2.03 ± 1.23	2.26 ± 1.24	0.007
Phosphorus (mmol/D)	8.34 ± 4.91	9.67 ± 5.89	0.001
Uric acid (μmol/D)	1474.15 ± 711.88	1639.50 ± 792.70	0.002

To further determine whether the clinical profile of patients with positive urine cultures varied according to bacterial species, we subdivided these patients into patients positive for E. coli and patients positive for other bacterial isolates, and compared both groups with patients with negative urine cultures (**Supplementary Table 1**). Among the 487 patients with culture positive CaOx stones, 162 were positive for E. coli and 325 were positive for other bacterial isolates. Compared with patients with negative urine cultures, patients positive for E. coli were older and showed a marked female predominance. They also had lower serum uric acid levels, a higher proportion of renal stones, larger maximum stone diameter, and lower urinary excretion of chloride, sodium, calcium, phosphorus, and uric acid measured over 24 hours. In contrast, patients positive for other bacterial isolates differed from patients with negative urine cultures mainly in age, sex distribution, hypertension prevalence, and stone location. Although the other bacteria group also showed numerically larger stone diameter and lower urinary excretion of several ions, these differences did not reach significance in pairwise comparisons after Bonferroni correction. These findings suggest that culture positive CaOx stones are clinically heterogeneous across bacterial species. Because E. coli was a frequent isolate in patients with culture positive CaOx stones and has been linked to CaOx stones in previous studies, we selected E. coli as a clinically relevant representative organism for the experimental arm.

We further performed multivariable logistic regression analyses to evaluate whether urine culture status remained associated with host and stone factors after adjustment (**Supplementary Table 2**). After adjustment for age, sex, BMI, hypertension, diabetes, stone laterality, stone location, and maximum stone diameter, urine culture positivity remained associated with female sex, renal stone location, renal and ureteral stone location, and larger maximum stone diameter. In subgroup models by bacterial category, E. coli positive urine culture was strongly associated with female sex and was also associated with renal stone location and larger maximum stone diameter. Other bacteria positive urine culture was associated with female sex, hypertension, renal stone location, renal and ureteral stone location, and larger maximum stone diameter. These adjusted associations indicate that the clinical differences among urine culture subgroups are strongly linked to host characteristics and stone related factors, rather than being attributable to bacterial species alone.

### 3.4 *Escherichia coli*-derived OMVs were successfully characterized, internalized by HK-2 cells, and reduced cell viability

**Figure 2:**
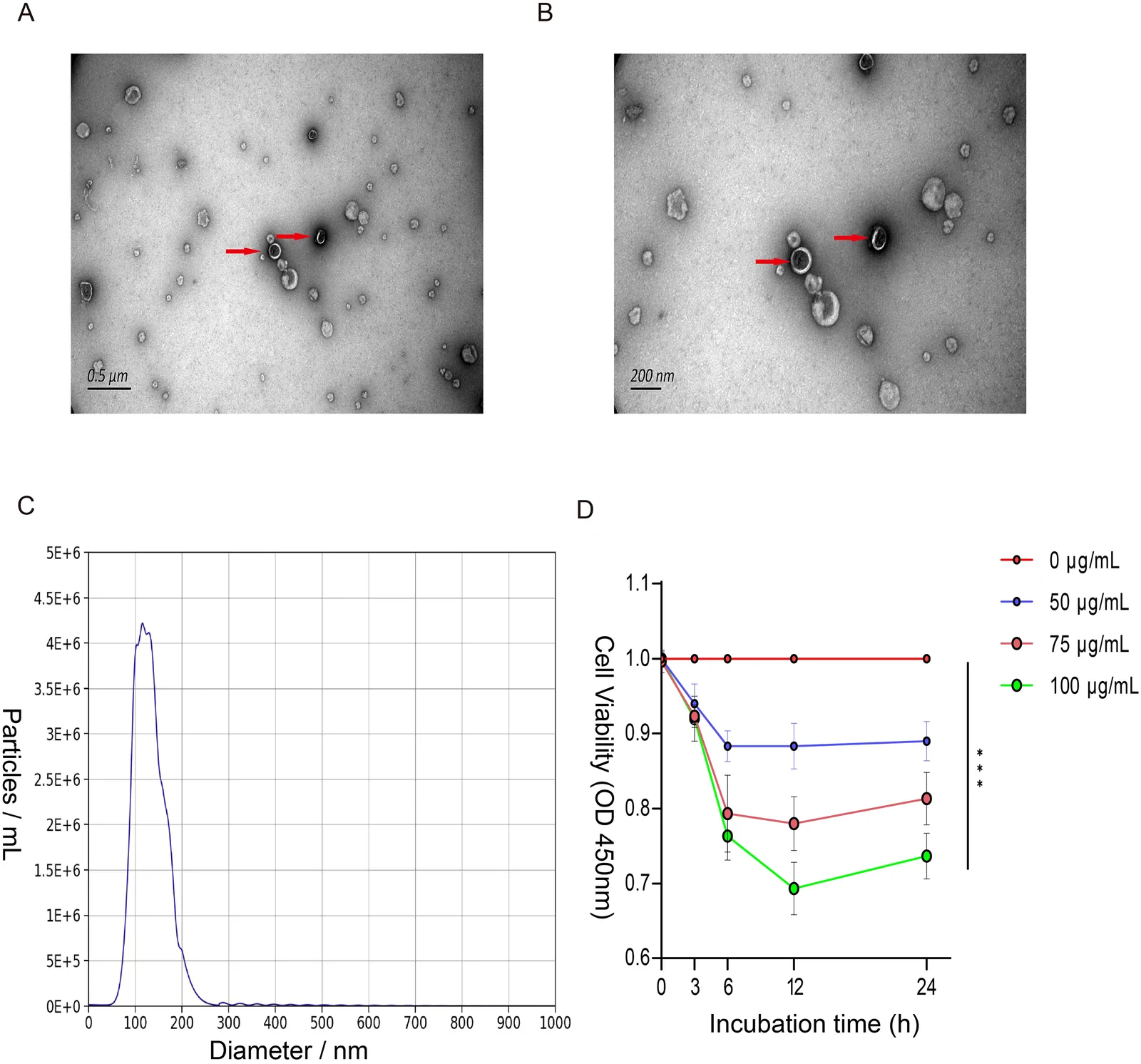
**(A, B)** Representative transmission electron microscopy images of purified OMVs showing nanosized vesicular structures (red arrows). **(C)** Nanoparticle tracking analysis showing the size distribution of purified OMVs. **(D)** CCK-8 assay showing the viability of HK-2 cells treated with *Escherichia coli*-derived OMVs at 0, 50, 75, or 100 μg/mL for 3, 6, 12, or 24 h. Cell viability was normalized to the untreated control group (N = 3). Data are presented as mean ± SD.

Purified *Escherichia coli*-derived outer membrane vesicles (OMVs) were characterized by transmission electron microscopy and nanoparticle tracking analysis. Transmission electron microscopy showed typical nanosized round vesicular structures, and nanoparticle tracking analysis confirmed that the purified particles were mainly distributed within the expected nanoscale range (**Figures 2A–C**).

The effect of OMVs on HK-2 cell viability was assessed by CCK-8 assay. Exposure to *Escherichia coli*-derived OMVs reduced HK-2 cell viability in a dose- and time-dependent manner, with more obvious inhibitory effects observed at higher concentrations and longer incubation times (**Figure 2D**). Based on the concentration- and time-response results, 100 μg/mL OMVs for 12 h was selected as a reproducible injury-inducing condition that preserved sufficient adherent cells for subsequent oxidative stress and COM crystal adhesion assays.

**Figure 3:**
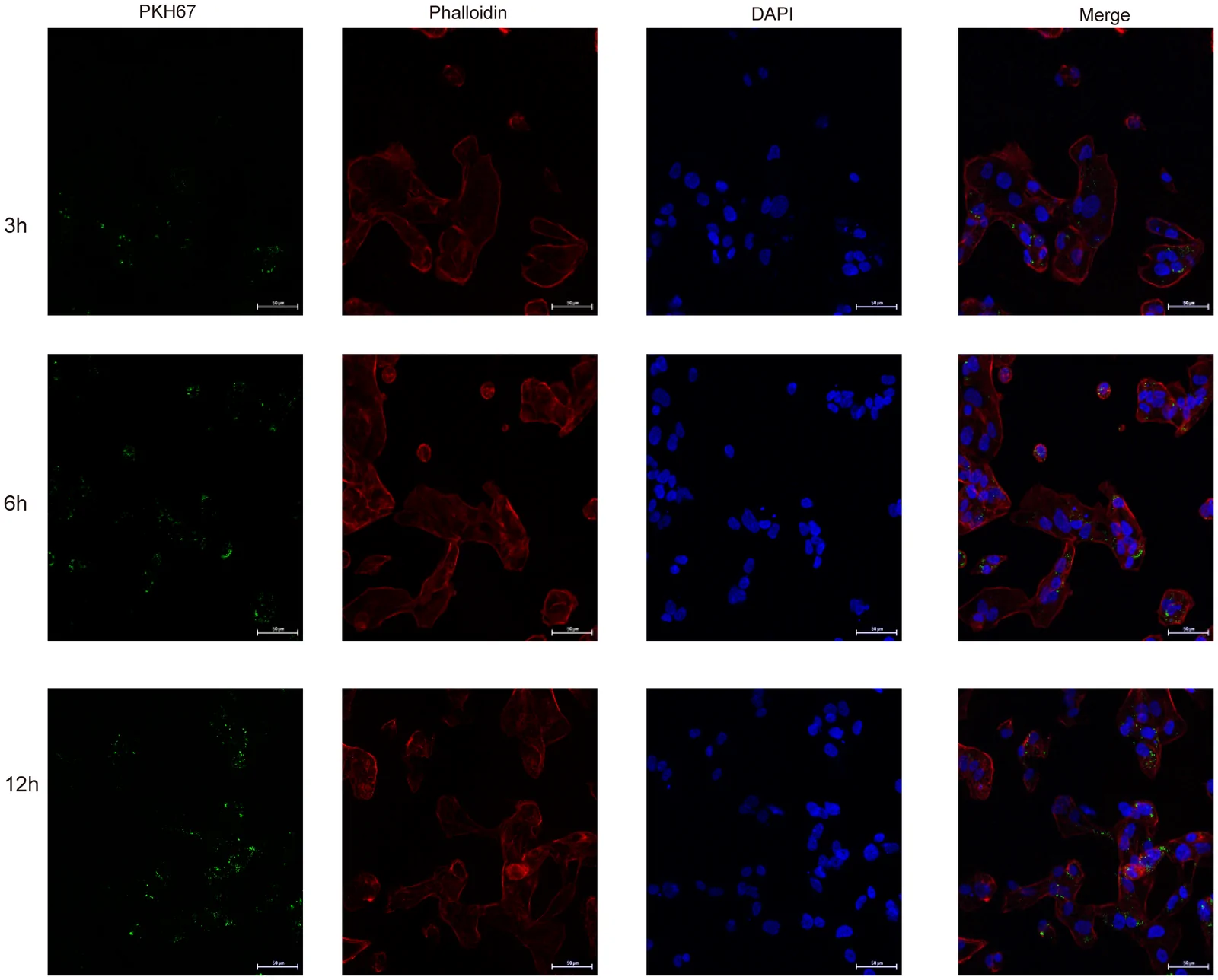
Representative confocal images of HK-2 cells exposed to PKH67-labeled *E. coli*-derived OMVs for 3, 6, and 12 h. Green fluorescence indicates PKH67-labeled OMVs, red indicates F-actin stained with phalloidin, and blue indicates nuclei stained with DAPI. Dye-only control images and quantitative analysis of PKH67-positive area are shown in **Supplementary Figures 1** and **S2**.

To determine whether these vesicles could interact directly with renal tubular epithelial cells, PKH67-labeled OMVs were incubated with HK-2 cells and examined by confocal microscopy. Intracellular punctate green fluorescence was observed in HK-2 cells after exposure to PKH67-labeled OMVs for 3, 6, and 12 h (**Figure 3**). To exclude nonspecific fluorescence from free PKH67 dye or dye aggregates, a dye-only control processed using the same labeling, quenching, and washing procedures in the absence of OMVs was included. The dye-only control showed only weak residual background fluorescence (**Supplementary Figure 1**). Quantitative analysis showed that the PKH67-positive area increased over time in the PKH67-labeled OMV group, whereas no significant time-dependent increase was observed in the dye-only control group (**Supplementary Figure 2**). These findings support time-dependent uptake of PKH67-labeled OMVs by HK-2 cells.

### 3.5 *Escherichia coli*-derived OMVs induced oxidative stress in HK-2 cells

**Figure 4:**
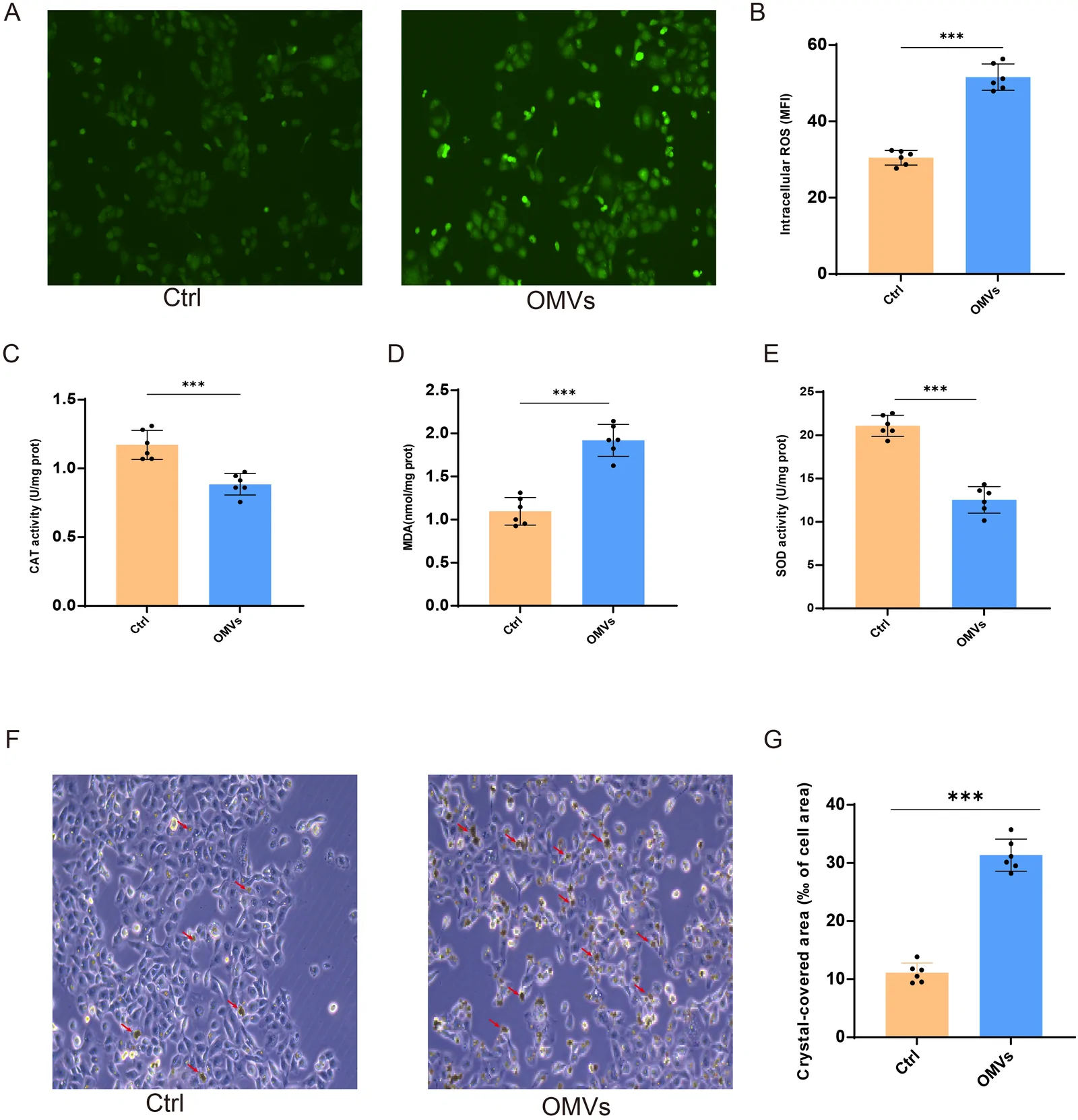
**(A)** Representative fluorescence images of intracellular reactive oxygen species (ROS) in control and OMV-treated HK-2 cells detected by DCFH-DA staining after 12 h of treatment. **(B)** Quantification of intracellular ROS fluorescence intensity. Data are presented as mean ± SD (n = 6). **(C–E)** Catalase (CAT) activity, malondialdehyde (MDA) content, and superoxide dismutase (SOD) activity in control and OMV-treated HK-2 cells. Data are presented as mean ± SD (n = 6). **(F)** Representative bright-field images of calcium oxalate monohydrate (COM) crystal adhesion to HK-2 cells in the control and OMV-treated groups; red arrows indicate adherent crystals. **(G)** Quantification of crystal-covered area, expressed as the percentage of crystal area relative to cell area. Data are presented as mean ± SD (n = 6). “***” indicates P < 0.001.

To investigate whether OMV exposure caused oxidative injury in renal tubular epithelial cells, intracellular ROS and oxidative stress-related biochemical parameters were evaluated. DCFH-DA staining demonstrated stronger intracellular ROS fluorescence in OMV-treated HK-2 cells than in control cells, and quantitative analysis confirmed a significant increase in ROS fluorescence intensity after OMV treatment (**Figures 4A, B**).

Consistently, OMV-treated cells showed decreased catalase (CAT) activity and superoxide dismutase (SOD) activity, together with increased malondialdehyde (MDA) content, compared with control cells (**Figures 4C–E**). These findings indicate that *Escherichia coli*-derived OMVs induce oxidative stress and impair antioxidant defenses in HK-2 cells.

### 3.6 *Escherichia coli*-derived OMV exposure was associated with increased COM crystal adhesion to HK-2 cells

Because epithelial injury is a critical determinant of crystal retention, we next examined whether HK-2 cells exposed to OMVs showed altered calcium oxalate monohydrate (COM) crystal adhesion under the same injury-inducing exposure condition. Bright-field microscopy revealed more adherent COM crystals on the surface of OMV-treated HK-2 cells than on control cells (**Figure 4F**). Quantitative analysis further showed that the crystal-covered area was significantly increased following OMV treatment (**Figure 4G**). These data indicate that HK-2 cells exposed to *E. coli*-derived OMVs under an injury-inducing condition exhibited increased COM crystal adhesion.

## 4 Discussion

In the present study, we combined a clinical analysis organized in three layers with *in vitro* functional experiments to investigate whether bacterial factors may contribute to calcium oxalate (CaOx) stone disease beyond classical infection stones. In the first layer, we compared CaOx, infection, and uric acid stones to define the clinical features and measured urinary biochemical parameters of CaOx stones relative to other major stone phenotypes. In the second layer, we focused on the CaOx cohort and compared patients with positive and negative urine cultures to examine whether urine culture positivity was associated with stone location, maximum stone diameter, and urinary biochemical parameters measured over 24 hours. In the third layer, among patients with CaOx stones and positive urine cultures, we further compared patients positive for E. coli with those positive for other bacteria and with culture negative patients to explore whether the clinical profile varied according to bacterial species. Although CaOx nephrolithiasis is traditionally viewed as a predominantly metabolic disorder, accumulating evidence suggests that bacteria may also participate in calcium stone disease through mechanisms other than urease activity and urinary alkalinization, including direct interactions between crystals and bacteria, biofilm effects, and host inflammatory responses (Barr-Beare et al., 2015; Schwaderer and Wolfe, 2017).

Using this three-layer design, we found that CaOx, infection, and uric acid stones differed in demographic features, comorbidities, stone characteristics, urine culture positivity, and measured 24-hour urinary biochemical parameters. Within the CaOx cohort, patients with positive urine cultures had a higher proportion of renal stones and a larger maximum stone diameter despite lower urinary excretion of several ions over 24 hours. Further analysis by bacterial species showed that patients positive for *E. coli* differed from culture negative patients across host, stone, and urinary biochemical features, including older age, female predominance, lower serum uric acid, a higher proportion of renal stones, larger maximum stone diameter, and lower urinary excretion of chloride, sodium, calcium, phosphorus, and uric acid over 24 hours. In contrast, the pooled other bacteria group showed a partially overlapping but less uniform pattern, with differences mainly in age, sex distribution, hypertension prevalence, and stone location. Multivariable analyses further showed that urine culture status, including E. coli positive urine culture, remained associated with measured host and stone factors after adjustment, particularly sex, renal stone location, and larger maximum stone diameter. These findings suggest that culture positive CaOx stones are clinically heterogeneous and provide the rationale for examining E. coli as a representative organism in the experimental arm. However, they also indicate that the observed E. coli associated phenotype cannot be attributed to bacterial species alone, and the clinical data cannot establish whether E. coli contributed directly to this phenotype or instead reflected host and stone factors associated with bacterial colonization or persistence. This interpretation is further complicated by the marked female predominance in the E. coli-positive subgroup and by the reliance on admission urine culture. Because repeated urine cultures, catheterized urine specimens, stone cultures, and stone microbiome sequencing were not available for all patients, the extent to which E. coli positivity reflected persistent urinary or stone-associated bacterial presence rather than transient colonization or sampling-related contamination could not be determined.

The decision to focus on E. coli was also supported by previous work from Zhong et al (Zhong et al., 2021), who analyzed urinary and stone E. coli isolates from patients with CaOx stones and found high similarity between paired urinary and stone isolates from the same patient in antimicrobial susceptibility, phylogenetic grouping, virulence gene profiles, and genomic relatedness. To provide a reproducible and clinically relevant UPEC model for the experimental arm, we used E. coli CFT073/WAM2267 (ATCC 700928), a genome sequenced UPEC reference strain originally isolated from blood and urine from a woman with acute pyelonephritis. Compared with nonpathogenic laboratory E. coli strains, CFT073 is more appropriate for modeling bacterial products released by urinary pathogenic E. coli. Nevertheless, CFT073 represents a single pyelonephritis-associated UPEC background and cannot capture the full genetic, phenotypic, and functional heterogeneity of E. coli isolates from patients with CaOx stones. Clinical urinary or stone-derived E. coli isolates may differ from CFT073 in phylogenetic background, virulence gene repertoire, antimicrobial resistance, surface antigen expression, OMV yield, OMV cargo composition, and biological activity. These strain-level differences may influence the magnitude and pattern of OMV-induced epithelial responses, and future studies using patient-derived urinary and stone isolates are therefore needed to determine the generalizability of these findings across clinically relevant E. coli strains. On the basis of this clinical and experimental rationale, we used E. coli derived outer membrane vesicles (OMVs) as representative bacterial products, because OMVs are recognized mediators of interactions between host cells and bacteria and can deliver lipopolysaccharide, proteins, and other cargo related to virulence into host cells independently of intact bacteria (Chen et al., 2023; Luo et al., 2025). We found that these OMVs could be internalized by HK-2 cells, reduce cell viability, induce oxidative stress, and enhance calcium oxalate monohydrate crystal adhesion. Given that oxidative injury and inflammation are closely linked to crystal retention and progressive stone formation in the kidney (Liu et al., 2022), these findings support the possibility that bacterial signals may contribute to an epithelial microenvironment that favors crystal retention in a subset of patients with CaOx stone disease.

This interpretation is supported by experimental studies on E. coli in CaOx stone disease. Barr-Beare et al (Barr-Beare et al., 2015). provided proof-of-concept that bacteria may worsen CaOx crystal disease by showing that kidney stones harbor Enterobacteriaceae, that E. coli aggregates around calcium oxalate monohydrate (COM) crystals, and that uropathogenic E. coli infection in mice increases renal CaOx deposition while augmenting innate immune activation and stone matrix protein expression. Moreover, pre-existing CaOx deposition increased bacterial burden in murine pyelonephritis, suggesting a bidirectional relationship between bacterial persistence and CaOx crystal disease. Consistent with this, Kanlaya et al (Kanlaya et al., 2019). showed that E. coli flagellum promotes CaOx crystallization, crystal growth, aggregation, and crystal-cell adhesion, indicating that specific bacterial factors can directly facilitate a crystal-retentive microenvironment. Together, these studies provide a strong rationale for focusing on E. coli in CaOx stone disease.

However, most previous studies have focused on intact bacteria or bacterial surface-associated structures. This leaves open the possibility that bacteria-related effects in CaOx stone disease may also be mediated by cell-free bacterial products rather than requiring persistent colonization by whole organisms. In this context, outer membrane vesicles (OMVs) represent a plausible alternative mechanism. As nanosized vesicles released by Gram-negative bacteria, OMVs can deliver lipopolysaccharides, proteins, and other bioactive cargo to host cells independently of intact bacteria. This concept is supported by studies in other renal and inflammatory settings. For example, Chen et al (Chen et al., 2023). showed that gut microbiota-derived OMVs could mediate tubulointerstitial inflammation in diabetic kidney disease, highlighting the ability of bacterial vesicles to act directly on renal tubular cells. More recently, Háček et al (Háček et al., 2025). reported that enterohemorrhagic E. coli O157 OMVs induced renal tubular injury and acute kidney failure in mice, further supporting the pathogenic potential of E. coli-derived OMVs in the kidney. Therefore, we reasoned that OMVs may represent a biologically relevant cell-free effector through which E. coli-associated bacterial signals may modify the CaOx stone microenvironment, particularly in patients without sustained overt infection.

Our *in vitro* findings provide functional support for this interpretation. We found that *E. coli*-derived OMVs were internalized by HK-2 cells in a time-dependent manner and were associated with reduced cell viability, increased intracellular ROS, decreased SOD and CAT activity, increased MDA content, and enhanced COM crystal adhesion. These findings are mechanistically relevant because recent reviews have consistently emphasized that CaOx stone formation depends not only on crystal nucleation, growth, and aggregation, but also on crystal–cell interactions, epithelial injury, and crystal retention within the renal tubules. Shi et al (Shi et al., 2025). summarized that urinary proteins, particularly matrix proteins, participate in multiple steps of CaOx stone formation, including crystal adhesion, whereas Wan et al (Wan et al., 2026). further highlighted that crystal–cell interactions, oxidative stress responses, inflammatory signaling, and the expression of cell adhesion molecules are closely integrated within the stone-promoting microenvironment. In parallel, Bai et al (Bai et al., 2025). and Liang et al (Liang et al., 2025). reviewed accumulating evidence that excessive ROS and pro-inflammatory signaling pathways, including NLRP3, NF-κB, and MAPK, contribute to renal tubular epithelial injury and favor crystal deposition and adhesion, whereas anti-inflammatory pathways may suppress crystal adhesion and oxidative damage. Against this background, OMV-induced oxidative stress accompanied by increased COM crystal adhesion suggests a plausible mechanism by which bacterial signals may amplify epithelial injury and promote a crystal-retentive microenvironment. Uromodulin, also known as Tamm–Horsfall protein, may represent another potential link between tubular epithelial injury, host defense, and crystal retention. As an abundant urinary glycoprotein, uromodulin participates in the regulation of calcium oxalate crystallization, growth, aggregation, crystal-cell adhesion, and matrix invasion (Noonin et al., 2022). It also contributes to host defense against uropathogens by binding type 1 fimbriated *E. coli* and preventing bacterial attachment to urothelial receptors (Pak et al., 2001). Oxidative modification of uromodulin has been reported to alter its CaOx-modulating activity, particularly by promoting calcium oxalate crystallization and growth, suggesting a potential connection between OMV-induced oxidative stress and altered urinary crystal regulation (Chaiyarit and Thongboonkerd, 2022). Because uromodulin expression, modification, and function were not assessed in the present study, future work should determine whether *E. coli*-derived OMVs influence uromodulin expression, modification, or function during CaOx crystal retention.

Taken together, the clinical and experimental findings provide complementary evidence that bacterial signals associated with E. coli may be relevant to a subset of CaOx stone disease, while also defining the limits of this interpretation. Clinically, patients positive for E. coli showed a broader pattern of differences from culture negative patients than did the pooled other bacteria group, involving host characteristics, stone location, larger maximum stone diameter, and urinary biochemical parameters measured over 24 hours. Experimentally, OMVs from E. coli were able to induce several epithelial changes relevant to stone formation, including reduced HK-2 cell viability, increased oxidative stress, and enhanced COM crystal adhesion. When considered together with the evidence discussed above, our findings support the hypothesis that products released by E. coli may influence the CaOx stone microenvironment in some patients. However, these data should be viewed as supporting a biologically plausible hypothesis rather than establishing that E. coli independently drives the observed clinical phenotype.

Several limitations should be acknowledged. First, the clinical component was retrospective, single-center, and based on urine cultures obtained at hospital admission. Therefore, the clinical analysis captures the association between contemporaneous bacteriuria and CaOx stone phenotype, but cannot resolve the temporal sequence between bacterial exposure and stone development. Admission urine culture may not reflect bacterial signals present during earlier stone nucleation or growth, nor the microbial composition within stones or the local stone microenvironment. Accordingly, whether bacteriuria precedes, accompanies, or follows differences in stone location and maximum stone diameter remains unresolved in the clinical cohort. Second, the metabolic characterization of CaOx stone formers was incomplete. In particular, 24-hour urinary oxalate and citrate were not available because these measurements were not routinely performed at our institution during the study period. This is clinically relevant because urinary oxalate is a major driver of CaOx supersaturation, whereas urinary citrate is an important endogenous inhibitor of CaOx crystallization. Therefore, the urinary biochemical findings in this study should be interpreted as differences in measured 24-hour urinary parameters rather than as a complete metabolic assessment of CaOx stone risk. We cannot exclude the possibility that unmeasured oxalate or citrate levels contributed to the observed differences between urine culture-positive and culture-negative CaOx stone patients. Third, the *E. coli*-positive subgroup showed a marked female predominance. Although sex was included as a covariate in the multivariable models, non-catheterized midstream urine collection may be more susceptible to contamination from periurethral or perineal flora in female patients. Because repeated urine cultures, catheterized urine cultures, stone cultures, and stone microbiome sequencing were not available for all patients, we could not fully quantify or exclude the contribution of contamination or distinguish true bacteriuria from contamination in every case. This limitation may have affected the observed associations between E. coli positivity and clinical stone features. Fourth, although multivariable models adjusted for age, sex, BMI, hypertension, diabetes, stone laterality, stone location, and maximum stone diameter, residual confounding remains possible. Information on prior antibiotic exposure, recurrent urinary tract infection history, menopausal status, hormonal factors, and immune status was not available in this retrospective dataset. Fifth, the other bacteria group comprised taxonomically and biologically diverse organisms with different Gram staining characteristics, urease activity, and pathogenic potential. Therefore, comparisons between patients positive for E. coli and those positive for other bacteria should be regarded as exploratory species level comparisons rather than direct evidence of species specific clinical effects. Sixth, the *in vitro* experiments used E. coli CFT073/WAM2267 (ATCC 700928), a genome sequenced UPEC reference strain, rather than patient derived urinary or stone isolates. Although this strain is clinically relevant to urinary infection, it does not represent the full heterogeneity of E. coli isolates in CaOx stone disease. Accordingly, the present study cannot determine which specific OMV cargo or host signaling cascade accounts for the observed effects on oxidative stress and crystal adhesion. Because LPS is an intrinsic component of Gram-negative bacterial OMVs, we also cannot determine whether these effects were mediated predominantly by intact vesicles as delivery structures, OMV-associated LPS, or other vesicle-associated cargo. Therefore, our findings should be interpreted as evidence that *E. coli*-derived OMVs have biological activity in inducing stone-relevant epithelial phenotypes, rather than as proof of an OMV-specific effect independent of LPS or other bacterial products. In addition, the OMV concentration used in the functional assays was selected to establish a reproducible *in vitro* epithelial injury model, whereas urinary OMV concentrations in patients with CaOx stones remain unknown. Therefore, these experiments support the biological plausibility of OMV-mediated epithelial injury and crystal adhesion, but do not establish that the same exposure level occurs in the urinary tract *in vivo*. Future studies using purified LPS, heat-inactivated or cargo-disrupted OMVs, OMVs from non-pathogenic or genetically modified strains, clinical *E. coli* isolates, stone-associated isolates, longitudinal urine and stone microbiological sampling, OMV cargo profiling, and pathway-intervention experiments are needed to distinguish the relative contributions of vesicle structure, LPS, and other OMV-associated components. Seventh, the COM crystal adhesion assay was performed under the same OMV exposure condition that reduced HK-2 cell viability. Therefore, the increased crystal-covered area may partly reflect OMV-induced epithelial injury, including changes in cell morphology, membrane integrity, or surface properties, rather than a direct adhesion-specific effect. Future studies using sub-cytotoxic OMV exposure, shorter incubation times, viability-matched conditions, or injury-blocking interventions will be needed to determine whether OMVs enhance crystal adhesion independently of epithelial injury. Finally, the clinical and experimental components of this study remain mechanistically indirect. Although the clinical analysis identified an *E. coli*-associated CaOx stone phenotype and the *in vitro* experiments showed that exposure to *E. coli*-derived OMVs was associated with stone-relevant epithelial injury, we did not directly measure OMVs in patient urine, stone matrices, or upper urinary tract samples. We also did not assess patient-level biomarkers of tubular oxidative injury or crystal-retention biology that could link bacteriuria to the epithelial phenotypes observed *in vitro*. Therefore, the clinical and experimental findings should be interpreted as complementary evidence supporting biological plausibility, rather than as direct evidence that OMVs mediate the observed clinical phenotype in patients.

In summary, urine culture positive CaOx stones, particularly those associated with E. coli, showed a clinically recognizable profile characterized by specific host and stone features. The adjusted analyses indicate that these differences are closely linked to measured host and stone factors, especially sex, renal stone location, and larger maximum stone diameter. The clinical data alone cannot determine whether bacteriuria is a contributor to, a consequence of, or a marker of the stone environment. However, the *in vitro* data showed that exposure to *E. coli*-derived OMVs was associated with OMV internalization, oxidative stress, and enhanced COM crystal adhesion in renal tubular epithelial cells, providing biological plausibility for a role of bacterial signals in modifying the CaOx stone microenvironment.

## 5 Conclusion

Our clinical findings suggest that urine culture positive calcium oxalate stones, particularly those associated with Escherichia coli, are associated with a recognizable profile involving female predominance, renal stone location, and larger maximum stone diameter. Because the clinical analysis was retrospective and based on urine culture at a single time point, these associations cannot determine whether bacteriuria preceded, accompanied, or followed the observed stone phenotype. To further examine the biological plausibility of bacterial involvement, we evaluated OMVs from E. coli *in vitro* and found that they were internalized by HK-2 cells and were associated with reduced cell viability, oxidative stress, and enhanced calcium oxalate monohydrate crystal adhesion. Together, these findings support a potential contributory role of bacterial factors in calcium oxalate stone pathogenesis and provide biological plausibility for *E. coli*-derived OMVs as cell-free bacterial effectors that may link bacteriuria to tubular epithelial injury and crystal retention.

## Data Availability

The original contributions presented in the study are included in the article/**Supplementary Material**. Further inquiries can be directed to the corresponding author.
