# Supplementary material for: Clinical features of Escherichia coli positive calcium oxalate stones and effects of outer membrane vesicles on tubular epithelial injury and crystal adhesion
Source: Front Cell Infect Microbiol. 2026 Aug 20;16:1859506. doi: 10.3389/fcimb.2026.1859506 (PMC13537981; doi:10.3389/fcimb.2026.1859506)
Supplement: Supplementary file 1 [file SupplementaryFile1.docx]

| Supplement table 1. Comparison among patients with calcium oxalate stones with *Escherichia coli*-positive urine culture, other bacteria-positive urine culture, and negative urine culture. | | | | |
| --- | --- | --- | --- | --- |
|  | *E. coli*-positive | Other bacteria-positive | Urine culture-negative | P-value |
| Patients | 162 | 325 | 790 |  |
| Age | 53.8±13.4 ^b^ | 51.2±14.8 ^c^ | 49.5±13.1 | 0.001 |
| Sex |  |  |  | 0.001 |
| Male(n) | 41 (25.3%) ^a, b^ | 215 (66.2%) ^c^ | 633 (80.1%) |  |
| Female(n) | 121 (74.7%) | 110 (33.8%) | 157 (19.9%) |  |
| Body Mass Index (kg/m²) | 24.87±4.05 | 25.61±5.11 | 25.21±3.77 | 0.157 |
| Underlying Diseases |  |  |  |  |
| Hypertensive | 41 (25.3%) ^a^ | 118 (36.3%) ^c^ | 227 (28.7%) | 0.015 |
| Diabetes | 28 (17.3%) | 53 (16.3%) | 114 (14.4%) | 0.546 |
| Biochemical testing |  |  |  |  |
| Creatinine (μmol/L) | 85.62±49.30 | 93.13±79.89 | 88.75±70.36 | 0.488 |
| Uric Acid (μmol/L) | 339.97±91.79 ^b^ | 358.52±95.27 | 376.96±96.84 | <0.001 |
| eGFR (mL/min/1.73 m²) | 89.32±81.22 | 88.42±29.11 | 91.32±24.13 | 0.472 |
| Parathyroid Hormone (pg/mL) | 60.47±106.64 | 62.70±74.60 | 56.87±58.42 | 0.508 |
| Serum Calcium (mmol/L) | 2.24±0.15 | 2.25±0.16 | 2.27±0.14 | 0.050 |
| Stone character |  |  |  |  |
| Stone laterality |  |  |  | 0.190 |
| Unilateral | 115 (71.0%) | 244 (75.1%) | 612 (77.5%) |  |
| Bilateral | 47 (29.0%) | 81 (24.9%) | 178 (22.5%) |  |
| Location of stones |  |  |  | <0.001 |
| Renal | 126 (77.8%) ^b^ | 243 (74.8%) ^c^ | 513 (64.9%) |  |
| Ureteral | 17 (10.5%) | 38 (11.7%) | 177 (22.4%) |  |
| Renal and Ureteral | 19 (11.7%) | 44 (13.5%) | 100 (12.7%) |  |
| Maximum stone diameter (mm) | 29.68±20.26 ^b^ | 28.96±24.02 | 23.36±16.91 | <0.001 |
| 24-hour urinalysis |  |  |  |  |
| Urine pH | 6.29±0.44 | 6.37±0.51 | 6.20±0.58 | 0.092 |
| Volume (L/D) | 2.09±0.85 | 2.10±0.82 | 1.99±0.73 | 0.147 |
| Chlorine (mmol/D) | 62.15±28.58 ^b^ | 67.39±31.69 | 70.69±32.70 | 0.029 |
| Sodium (mmol/D) | 75.93±34.25 ^b^ | 83.58±36.16 | 87.49±38.47 | 0.010 |
| Potassium (mmol/D) | 16.25±7.72 | 16.57±7.99 | 17.64±8.20 | 0.107 |
| Calcium (mmol/D) | 1.94±1.15 ^b^ | 2.08±1.27 | 2.26±1.24 | 0.016 |
| Phosphorus (mmol/D) | 7.41±4.47 ^b^ | 8.79±5.06 | 9.67±5.89 | <0.001 |
| Uric acid (μmol/D) | 1325.39±573.35 ^a, b^ | 1546.00±760.68 | 1639.50±792.70 | <0.001 |

Superscript letters indicate significant pairwise differences after Bonferroni correction: a, *E. coli*-positive vs other bacteria-positive; b, *E. coli*-positive vs urine culture-negative; c, other bacteria-positive vs urine culture-negative (all *P* < 0.0167).

| Supplementary Table 2. Multivariable logistic regression analyses of factors associated with urine culture status among patients with calcium oxalate stones. | | | | | | |
| --- | --- | --- | --- | --- | --- | --- |
| Variable | Urine culture positive vs negative, aOR (95% CI) | P-value | E. coli positive vs negative, aOR (95% CI) | P-value | Other bacteria positive vs negative, aOR (95% CI) | P-value |
| Age, per 10 years | 1.07 (0.97–1.18) | 0.188 | 1.17 (0.99–1.38) | 0.059 | 1.03 (0.92–1.15) | 0.620 |
| Sex, female vs male | 3.75 (2.86–4.92) | <0.001 | 11.32 (7.38–17.36) | <0.001 | 2.22 (1.62–3.03) | <0.001 |
| BMI, per kg/m² | 1.03 (1.00–1.06) | 0.098 | 1.04 (0.98–1.09) | 0.177 | 1.02 (0.99–1.06) | 0.138 |
| Hypertension, yes vs no | 1.15 (0.86–1.53) | 0.359 | 0.59 (0.36–0.98) | 0.041 | 1.39 (1.02–1.91) | 0.038 |
| Diabetes, yes vs no | 0.96 (0.67–1.39) | 0.840 | 1.05 (0.59–1.88) | 0.872 | 0.94 (0.62–1.40) | 0.751 |
| Stone laterality, bilateral vs unilateral | 1.13 (0.84–1.52) | 0.431 | 1.50 (0.93–2.41) | 0.100 | 1.00 (0.72–1.40) | 0.980 |
| Stone location, renal vs ureteral | 1.95 (1.33–2.86) | <0.001 | 2.42 (1.24–4.70) | 0.009 | 1.82 (1.19–2.77) | 0.005 |
| Stone location, renal and ureteral vs ureteral | 1.86 (1.15–3.02) | 0.012 | 2.17 (0.94–5.00) | 0.069 | 1.84 (1.08–3.12) | 0.025 |
| Maximum stone diameter, per 10 mm | 1.12 (1.05–1.20) | 0.001 | 1.13 (1.01–1.26) | 0.033 | 1.11 (1.03–1.20) | 0.004 |

Note: Each model used urine culture negative patients as the reference group. Models were adjusted for age, sex, BMI, hypertension, diabetes, stone laterality, stone location, and maximum stone diameter. Age and maximum stone diameter were modeled per 10 year and per 10 mm increase, respectively. Reference categories were male sex, no hypertension, no diabetes, unilateral stones, and ureteral stones. aOR, adjusted odds ratio; CI, confidence interval; BMI, body mass index.
