# Supplementary material for: Clinical features of Escherichia coli positive calcium oxalate stones and effects of outer membrane vesicles on tubular epithelial injury and crystal adhesion
Source: Front Cell Infect Microbiol. 2026 Aug 20;16:1859506. doi: 10.3389/fcimb.2026.1859506 (PMC13537981; doi:10.3389/fcimb.2026.1859506)
Supplement: Supplementary file 2 [file SupplementaryFile2.docx]

Supplementary Figure S1. Dye-only control for the PKH67-labeled OMV uptake assay.

Representative confocal images of HK-2 cells incubated with the dye-only control for 3, 6, and 12 h. The dye-only control was prepared using the same PKH67 labeling, quenching, and washing procedures in the absence of OMVs. Green indicates PKH67 fluorescence, red indicates F-actin stained with phalloidin, and blue indicates nuclei stained with DAPI. Scale bars, 50 μm.

Supplementary Figure S2. Quantification of PKH67 fluorescence in the OMV uptake assay.

(A) Quantification of PKH67-positive area in HK-2 cells exposed to PKH67-labeled *E. coli*-derived OMVs for 3, 6, and 12 h.
(B) Quantification of PKH67-positive area in HK-2 cells incubated with dye-only control for 3, 6, and 12 h.
Data are presented as mean ± SD from three independent experiments. *P* < 0.05 (*), *P* < 0.001 (***); ns, not significant.
